# Supplementary material for: Are carbon emissions trading and green financial instruments synergistic? -Comprehensive quantitative research based on content analysis
Source: PLoS One. 2024 Mar 7;19(3):e0298601. doi: 10.1371/journal.pone.0298601 (PMC10919601; doi:10.1371/journal.pone.0298601)
Supplement: S1 Data — (ZIP) [file pone.0298601.s001.zip › Data/地级市Z_F工作报告.zip!/╕╩╦α/╠∞╦«╩╨/└┤╫╘╣1⁄2╓┌║┼í╛┬φ┐╦╩2╛▌═°í┐_2013.docx]

2012年工作回顾

2012年,在省委、省政府和市委的坚强领导下,我市坚持以科学发展观为指导,认真贯彻落实省第十二次、市第六次党代会精神,抢抓机遇,真抓实干,完成了市六届人大一次会议确定的各项目标任务。预计实现生产总值416亿元,增长13.6%;大口径财政收入71亿元,增长18%,其中一般预算收入23亿元,增长23.46%;财政支出162亿元,增长10.45%;社会消费品零售总额178.6亿元,增长19%;城镇居民人均可支配收入15009元,增长15%;农民人均纯收入3798元,增长16.3%。省政府考核的13项主要经济指标除4项与责任目标持平外,其余9项均高于责任目标。

项目工作成效显著。预计完成固定资产投资412亿元,增长41%;争取到国家及省上投资项目395项,总投资68.69亿元,落实投资12.96亿元;市列50个重大项目当年完成投资87.58亿元。

工业经济持续增长。预计规模以上工业企业实现增加值86亿元,增长16.5%;“10强50户”企业实现增加值68亿元,增长18%。

农村经济健康发展。预计实现农业增加值76亿元,增长7.5%。科技增粮措施有效落实,完成粮食播种467.61万亩,总产量116.7万吨,连续九年保持增长。

商贸旅游繁荣活跃。城乡市场体系建设进一步完善,“万村千乡”、农贸市场改造、农超对接等工程有序实施,新建、改扩建市场35处,新建和改造农贸市场8个,新增农家店220个。

城市建设步伐加快。区域性现代化大城市建设的思路更加完善,构架更加明晰,功能定位更加准确,新一轮城市总体规划修编工作全面启动。城市基础设施建设全面加强,历史文化名城保护力度不断加大,“三城联创”深入开展,五县县城和重点镇公共服务设施不断完善。

改革开放不断深化。农村各项改革深入推进;集体林权制度配套改革步伐加快;企业改制遗留问题得到妥善解决;城市管理综合行政执法体制改革深入推进;行政审批制度改革不断深化;以县级公立医院改革为主的医药卫生体制改革稳步推进;关中—天水经济区规划加快实施;对外影响力不断提升,举办了甘肃省公祭伏羲大典暨第23届中国天水伏羲文化旅游节等节庆活动;招商引资成果较大;对外贸易稳步增长。

社会事业协调发展。教育事业优先发展,“两基”成果进一步巩固;教育教学条件不断改善;科技创新步伐进一步加快;知识产权保护成效明显;“文化惠民”工程深入推进;文物和非物质文化遗产保护不断加强;文艺创作成果丰硕;全民健身活动广泛开展;广播影视事业快速发展;公共卫生进一步加强人口和计划生育、国土资源管理等工作取得新成效。

人民生活日益改善。省上确定的10项32件、市上确定的16件惠民实事全面完成。扶持高校毕业生就业3300人,新增城镇就业5.74万人,年末城镇登记失业率3.19%。为31723户妇女发放小额担保贷款18亿多元。城乡居民社会养老保险实现全覆盖。

民主法治全面加强。依法治市进程不断加快;政务公开不断加强,重大决策专家咨询、社会公示、听证、跟踪督查和新闻发布等制度得到有效落实;反腐倡廉深入推进;村务公开民主管理和监督工作积极开展;“平安天水”建设深入推进;矛盾纠纷排查调处力度不断加大;“安全生产年”和“打非治违”等活动深入开展;食品、药品、知识产权等专项整治和物价调控工作取得新成效;民族团结、宗教和顺、社会和谐的大局得到巩固和发展。

回顾过去的一年,政府工作虽然取得了一些新的成绩,但我们也清醒地看到,全市经济社会发展仍面临不少困难和问题,主要表现在:一是产业结构亟待优化升级,工业经济比重较低,第三产业发展不充分、不平衡。二是城镇化水平不高,推进城乡一体化的任务繁重。三是社会事业发展相对滞后,公共服务水平有待提升。四是社会保障体系不够健全,城乡居民收入水平依然较低,就业再就业压力较大。五是社会管理中还存在一些薄弱环节,影响社会和谐的因素仍然不少,维护稳定的任务依然艰巨。六是发展环境还不宽松,政风行风还需改进,政府职能还需加快转变。对于这些问题,我们将努力在实践中逐步加以解决。

2013年主要任务

2013年,是加快实施“十二五”规划的关键一年,也是为全面建成小康社会夯实基础的重要一年。当前,加快发展面临的困难和不确定因素尽管存在,但总体来看,我市正处在加快发展的黄金期、政策叠加的机遇期和奋力崛起的爬坡期。只要我们科学判断形势、准确把握机遇、坚持真抓实干,前进中遇到的困难和问题一定能够有效解决,转型跨越的目标一定能够按期实现。

政府工作的总体思路是:深入贯彻落实党的十八大和省第十二次、市第六次党代会精神,以科学发展观为指导,以全面建成小康社会为目标,坚持“关天经济区引领、国家级园区支撑、现代化城市带动、县区组团式推进”,全面实施“1135”发展战略、“十二五”发展规划和“3341”项目工程,牢牢把握“稳中求进”的工作总基调,着力深化改革开放,着力转变发展方式,着力优化产业结构,着力保障改善民生,推动经济持续健康发展和社会和谐稳定,努力把天水建成综合实力较强的区域性现代化大城市。

全市经济社会发展的主要预期目标是:生产总值增长14%,大口径财政收入增长19%,固定资产投资增长36%,规模以上工业增加值增长17%,社会消费品零售总额增长18%以上,城镇居民人均可支配收入增长16%以上,农民人均纯收入增长16.5%以上,居民消费价格指数控制在104%左右,城镇登记失业率控制在4%以内,人口自然增长率控制在7.3‰以下,万元生产总值综合能耗下降3.2%。

围绕上述目标,市政府将重点抓好以下八个方面的工作:

全面实施“3341”项目工程

始终把项目工作作为转型跨越发展的关键和着力点,突出主攻方向,聚焦着力重点,以项目拉动投资,以项目调整结构,以项目改善民生,为推动经济社会转型跨越、实现赶超进位提供动力。

提升项目谋划水平。按照“3341”项目工程的总体要求,围绕打造转型跨越发展平台、破解基础设施瓶颈制约、做大做强优势产业集群,研究国家产业政策及我市产业规划和项目布局,抢抓国内外产业转移等重大机遇,谋划和储备一批打基础、利长远的重大项目,并结合天水的资源优势、产业基础、生态条件,培育壮大战略新兴产业、特色优势产业、富民多元产业和区域首位产业,形成跨越发展的多元支撑。

加快重大项目建设。组织实施重点建设项目100个,抓好宝鸡至兰州铁路客运专线、天水至平凉铁路等重大项目建设,开工建设天水至武都铁路、天水至平凉高速公路等工程。推进天水机场迁建、曲溪城乡供水工程、陇县至甘谷高速公路、330千伏社棠输变电等项目前期工作进度,争取早日开工建设。

强化项目服务保障。坚持领导包抓项目制度,完善项目工作机制,科学分解目标任务,加大督促检查,确保重大项目建设顺利推进。强化协作配合,做好征地搬迁等保障服务工作,健全完善项目投融资体制机制,营造良好的项目建设环境。

推动工业经济转型升级

围绕“突出一个振兴、建设一区多园、发展六大集群、壮大县域经济”的思路,推进“12341”行动计划和“10强50户”工程,提升工业运行质量和效益。

加快开发区扩区增容。推进天水经济技术开发区扩区增容,把25个县区工业园纳入开发区统一管理,抓好县区工业园建设规划编制和优惠政策落实。调整完善开发区管理运行机制,加强基础配套设施建设,增强产业聚集功能,加快入园项目建设进度,强化协调服务,加快城区33户工业企业出城入园进程。

培育壮大工业产业集群。落实扶持工业经济发展各项优惠政策,培育壮大机械制造、电工电器、电子信息、医药食品、建筑材料、能源化工六大产业集群,尤其要突出装备制造业的区域首位产业地位,集中力量将其打造成为能够对地方经济起决定性带动作用的首位产业。

积极推进企业技术进步。实施质量振兴和商标战略,加快传统产业升级改造,推进信息技术与工业化深度融合,推动企业创新能力和名牌品牌建设,争创省级及以上技术中心2家、省级和国家级技术创新示范企业3家、中国驰名商标2件、省著名商标14件、甘肃名牌产品7件,不断增强企业核心竞争力。

大力扶持中小微企业发展。落实鼓励扶持非公经济和中小微企业发展的政策措施,在市场准入、土地使用等方面强化政策引导。健全完善融资等公共服务体系,逐步建立中小企业、个体工商户信贷服务创新机制和平台。加强中小微企业运行监测和协调服务,帮助企业解决好生产经营中的困难和问题,促进中小微企业和非公经济快速发展。

加强矿产资源开发利用。实施“三年地质找矿行动计划”,积极争取中央和省级地勘基金,鼓励、支持和引导社会资本投入地质找矿工作。按照矿区整装开发的思路,加大重点成矿区(带)深部、外围风险性勘查和危机矿山接替资源找矿工作力度,重点抓好麦积六丈山铷矿等矿产资源勘探开发。强化产业规划和项目对接,加快矿产资源加工转化,尽快形成工业经济新的增长点。

大力发展现代农业

把“三农”工作作为重中之重,以发展现代农业为方向,以加快推进农业产业化为重点,以现代农业示范区和农业农村科学发展示范点创建为抓手,促进农民增收、农业增效、农村繁荣。

发展壮大特色优势产业。按照“南林、北果、东牧、西菜”的产业布局,狠抓特色优势农产品示范区建设,打造果品产业“五个绿色基地”、蔬菜产业“一区两园三基地”、畜牧产业“四大养殖基地”。实施“百万亩优质果园提质增效工程”;实施“百万亩蔬菜标准化创建工程”;实施“百万头肉牛养殖工程”;实施“百户重点龙头企业建设工程”,在每个县区新发展2个以上有一定规模的农产品精深加工企业,市级以上龙头企业增加到140户以上。

不断提升农业科技水平。加快天水国家农业科技园区提质扩园工作,做大做强航天育种产业,争取众兴菌业尽快上市,促进园区产业融合发展。推进10个现代农业示范区建设,继续抓好麦积区颖川河流域综合开发等一批农业农村科学发展示范点创建,增强示范带动效应。推进地膜、种子、植保、沃土、农机五大科技增粮工程。

积极改善农业基础条件。组织实施好十大水利重点工程,抓好武山、秦州全国小型农田水利重点县和坡改梯试点县项目建设,加快渭河干支流堤防、山洪灾害防治等工程建设。实施“百万亩优质梯田建设”和甘肃东部重大土地整治等工程;加强农村沼气建设与服务管理等工作,实施“一池三改”3000户,提升沼气利用率。

加快推进新农村建设。实施“省列一县、市列一线七片”新农村建设片带推进工程、渭河川道城乡一体化百里长廊示范工程等八大工程,努力改变农村落后面貌。打好扶贫攻坚战,深入开展“联村联户、为民富民”行动,实施好六盘山片区区域发展与扶贫攻坚规划,全面启动“百万贫困人口减贫计划”,坚持整流域推进、片带式开发的扶贫开发模式,实现20万人减贫目标,加快农村脱贫致富步伐。
